# Experiences of colorectal cancer patients in Australia: a qualitative study on specialised nursing and supportive care

**DOI:** 10.1007/s00520-026-10333-6

**Published:** 2026-01-29

**Authors:** Karina T. Rune, Jared Ardern, Cindy Davis

**Affiliations:** 1 School of Health, University of the Sunshine Coast, Sippy Downs, QLD 4556 Australia; 2 School of Law and Society, University of the Sunshine Coast, Sippy Downs, QLD 4556 Australia

**Keywords:** Colorectal cancer, Survivorship, Cancer nursing, Coping strategies, Patient care

## Abstract

**Background:**

Colorectal cancer (CC) is the third most common cancer globally. Despite advances in treatment, patients often experience long-term psychological, physical, and social challenges during and after treatment. Specialised cancer care, including the role of cancer nurses, is critical in supporting patients throughout their treatment journey.

**Aims:**

This study aimed to explore the experiences of CC patients in Australia in navigating the healthcare system, coping with their diagnosis, and receiving support from specialised cancer nurses and support networks.

**Method:**

An exploratory qualitative design was used. Semi-structured interviews were conducted with nine CC patients (seven females, two males), aged 34–72 years. Interviews were analysed using reflexive thematic analysis.

**Results:**

Four overarching themes emerged. First, challenges navigating the healthcare system, identified participants’ frustrations with delays, miscommunication, and fragmented care. Second, emotional impact of a CC diagnosis and treatment, captured the shock, fear, and isolation experienced, particularly following sudden diagnoses. Third, value of specialised cancer nurses and support networks, highlighted the emotional reassurance and practical guidance provided by community-based nurses and support services. Fourth, physical and mental coping strategies included participants’ use of mindfulness, physical activity, and dietary changes to regain a sense of control.

**Conclusion:**

Specialised cancer nurses played a crucial role in enhancing patient care by addressing both medical and emotional needs. Improving communication, ensuring continuity of care, and providing personalised support are key recommendations for improving the healthcare experience of CC patients in Australia.

**Supplementary Information:**

The online version contains supplementary material available at 10.1007/s00520-026-10333-6.

## Introduction

Colorectal cancer (CC), also known as bowel cancer, is the third most common cancer worldwide and a leading cause of cancer-related morbidity and mortality [1]. In Australia, CC accounts for approximately 9.3% of all new cancer diagnosis and 10% of deaths from cancer, making it the second deadliest cancer after lung cancer [2], Cancer [3]. Despite advances in treatment, CC patients frequently experience ongoing symptoms and impairments in functioning, including physical, emotional, and social domains [4, 5]. Many patients are faced with managing these symptoms independently, highlighting the need for follow-up care that addresses long-term effects and provides targeted supportive interventions [5].

Australia’s healthcare system is a mixed public–private model, with universal coverage provided through Medicare and additional services funded via private insurance or out-of-pocket costs. Cancer care is delivered across both sectors, often involving complex navigation between hospitals, general practitioners, and specialist services. To address this complexity, national Optimal Care Pathways (OCPs) have been developed to standardise high-quality, coordinated cancer care across the country (Cancer [6, 7], Cancer [8]). Supportive care is a core principle of the OCPs and includes services addressing the physical, emotional, practical, and spiritual needs of individuals affected by cancer (Cancer [6, 7]). This includes symptom management, psychological support, rehabilitation, and assistance with daily living activities (Cancer [6, 7]). Specialised cancer nurses, such as Bowel Care Nurses, play a pivotal role in delivering supportive care. They provide patients with a consistent point of contact, assist in care coordination, manage treatment side effects, and offer psychosocial support throughout the cancer journey (Bowel Cancer [6, 7]). These nurses are particularly crucial in regional and remote areas, where access to comprehensive cancer services may be limited [9].

Cancer patients often report dissatisfaction with their quality of life (QoL), with concerns over body image, fatigue, sexual dysfunction, and sleep disturbances [10, 11]. Additionally, CC patients also struggle with the emotional toll of cancer, including stigma, loss, fear, and the long-term impact of their diagnosis [10, 12, 13]. In this context, specialised nurses can play an important role in offering personalised, holistic support that enhances cancer care [14]. While these psychosocial and physical challenges are experienced globally by CC patients, they are often compounded in Australia by geographical barriers. Individuals living in regional and remote areas may have limited access to specialised cancer support services, resulting in additional burdens during and after treatment [9]. This study is situated within the supportive care phase of the cancer care continuum, focusing on the lived experiences of patients navigating diagnosis, treatment, and survivorship. In this manuscript, we use the term ‘patients’ to refer to both individuals currently receiving treatment and those in post-treatment or survivorship phases. We retain the term ‘survivorship’ where it pertains to care models, systems, or literature focused on post-treatment care.

The biopsychosocial model provides a comprehensive framework for understanding CC patients’ experiences, emphasising the biological, psychological, and social factors involved in recovery. CC presents unique challenges compared to other cancers due to the physical, psychological, and social stigmas attached to bowel function and control [12, 15, 16]. Physically, treatment may cause fatigue, pain, and loss of bowel control, complicating recovery [17, 18]. Psychologically, patients are at increased risk of depression and stigmatisation, linked to stoma use, urinary symptoms, and reproductive health issues, which can negatively impact QoL [16, 19]. Social support from family, friends, and broader social networks also plays an important role in improving mental well-being and QoL for cancer patients [20, 21]. While extensive research has explored social support in breast and prostate cancers (e.g., [21–23]), CC remains under-researched in this area [24].

Coping with bowel cancer extends beyond the physical effects of the disease. Walshe et al. [25] found that peer support can foster emotional resilience and reduces isolation among cancer patients, including those with advanced CC. Community-based strategies and nurse-led clinics also play a vital role in addressing medical and emotional needs [26]. These integrated networks help patients manage the complexities of cancer survivorship [25, 26]. Additionally, patients often feel overwhelmed by the demands of the healthcare system, particularly during the early stages of diagnosis and treatment. Delays, miscommunication, and fragmented care contribute to increased stress and anxiety [11, 27, 28]. Clear communication and ongoing interaction with healthcare providers are central to a positive care experience, yet these areas often need improvement [29].

CC patients also report long-term challenges, such as fatigue, negative body image, and sexual dysfunction, often without adequate professional support [5, 30, 31]. Stigmatisation related to bowel function, such as the use of ostomy bags and concerns about incontinence, can lead to feelings of embarrassment and social withdrawal, further complicating recovery [13, 32]. Nutritional advice is frequently lacking, despite many CC patients reporting a need for personalised guidance throughout their recovery [33]. For patients with advanced CC, the journey is often prolonged and unpredictable, requiring ongoing, multidisciplinary support to manage both physical and psychosocial side effects [4, 15, 16].

Specialised cancer nurses play a pivotal role in bridging the gap between medical treatment and emotional support [34]. These nurses provide information, manage side effects, and offer emotional reassurance throughout the patient’s cancer journey [14, 21]. Nurse-led care models improve patient satisfaction and QoL by addressing both medical and emotional needs [26, 35]. By integrating psychological and social support with medical care, these nurses help patients manage the complex demands of CC [14, 21].

Despite growing recognition of the value of specialised cancer nursing, there remains limited empirical research examining how these nurses support CC patients throughout the treatment and survivorship phases, particularly within the Australian context. Existing studies tend to focus on general cancer populations or system-level evaluations, often overlooking the lived experiences of CC patients and how nurse-led support influences their psychological and social recovery. This study addresses this gap by exploring the perspectives of CC patients who received supportive care from specialised cancer nurses, in Australia. Specifically, it investigates (1) how a CC diagnosis impacts patients’ quality of life, including psychological and social challenges during and after treatment; and (2) what role specialised cancer nurses play in supporting patients through these phases.

## Methods

### Participants

Nine participants (seven females and two males) took part in the study, aged between 34 and 72 years (*M* = 52.44, *SD* = 14.59). No participants declined participation or withdrew from the study. All had been diagnosed with CC, and most had undergone or were undergoing treatment for CC. Table 1 provides a detailed breakdown of participant demographics, including cancer stage, years since diagnosis, and treatment history. Although the sample size is small, it aligns with the principles of reflexive thematic analysis, which prioritises depth, nuance, and the generation of rich, contextually situated insights over broad generalisability or data saturation [36, 37].

**Table 1:** Demographic information (*N* = 9)

Characteristics	*N* (%)
Gender	
Male	2 (22.20)
Female	7 (77.80)
Bowel cancer stage at diagnosis	
Stage 3	5 (55.50)
Stage 4	4 (44.40)
Treatment (current and past)	
Surgery	9 (55.50)
Chemotherapy	9 (55.50)
Radiation	4 (44.40)
Immunotherapy	
Hormone therapy	
Other	5 (55.50)
Years since diagnosis	
0–1	3 (33.33)
2–5	2 (22.22)
6–10	2 (22.22)
11–20	1 (1.11)
20 +	1 (1.11)

### Design and procedure

This study used an exploratory qualitative design aimed at understanding the lived experiences of CC patients in Australia. The research was guided by Braun and Clarke’s [38] six-phase framework for thematic analysis, which provides a systematic approach to identifying, analysing, and reporting patterns within qualitative data. The study followed the COREQ (Consolidated Criteria for Reporting Qualitative Research) checklist to enhance transparency and rigour in reporting qualitative research methods [39], see Supplementary Material).

Participants were recruited via social media and flyers distributed at a community cancer support centre. Participants were recruited using convenience sampling. Eligible individuals self-selected into the study after responding to flyers or social media posts. Inclusion criteria were as follows: (1) current or past diagnosis of CC, (2) age 18 or older, (3) currently residing in Australia, and (4) sufficient English proficiency to participate in a semi-structured interview. No additional exclusion criteria were applied. Demographic information was collected at the start of the interview, covering cancer stage at diagnosis, current cancer stage, treatment history, age, and gender.

The semi-structured interview questions included ten open-ended questions designed to explore participants’ experiences, challenges, and coping strategies related to bowel cancer, focusing on their journey through diagnosis and treatment, and the role of specialised care. The interview questions were drawn from previous research (e.g., [11, 28, 40]) and developed in consultation with experts in cancer care, including a specialist CC nurse, a psycho-oncology researcher, and a psychologist with experience in cancer research. Feedback from these experts led to revisions, including the addition of prompts to encourage broader, more detailed responses. Sample questions included, “Can you tell me about your cancer journey?” and “What have been your experiences with the bowel cancer nurse?” The full interview guide is available in the Supplementary Material.

All interviews were conducted by a male member of the research team, who was working as a counsellor at the time of the study, with a background in counselling and graduate-level psychology study. The interviewer also received additional training from the co-authors (including a clinical psychologist and a psycho-oncology expert) in qualitative interviewing and reflexive thematic analysis prior to data collection. There was no prior relationship between the interviewer and any of the participants before the study commenced. Prior to the interview, participants were provided with an information sheet outlining the interviewer’s role as a member of the research team and the purpose of the study. The interviewer had an interest in patient-centred cancer care and was aware of the potential for personal bias; reflexivity was used throughout data collection and analysis to minimise the influence of prior assumptions.

Due to COVID-19 restrictions in place during the study, interviews were conducted via phone or Zoom. This also ensured accessibility for participants living in rural or regional areas, reducing the burden of long-distance travel. Interviews lasted approximately 40 min. Participants did not receive compensation for taking part. No other individuals were present during the interviews, and no repeat interviews were conducted. Field notes were not taken; all interviews were audio recorded and transcribed verbatim. Transcripts were not returned to participants, and findings were not shared for participant checking. Verbal consent was obtained, and pseudonyms were used to ensure anonymity. Ethical approval was granted by the [*insert name*].

### Data analysis

Data analysis followed Braun and Clarke’s [38] six-phase reflexive thematic analysis. After transcription, the first step involved familiarising with the interview data by reviewing transcripts and taking notes on key points of interest, such as participant experiences with bowel cancer and the role of specialised care. The second step involved generating initial codes based on recurring language used by participants to describe their experiences, such as “how the nurse helped me” and “factors related to QoL.” Codes were iteratively developed as new patterns emerged during transcript review. In the third phase, codes were grouped into 12 initial themes that reflected common patterns in participant responses. Examples of early themes included “facing the unknown,” “need for support,” and “impact on work and family.” These preliminary themes were then reviewed and refined in the fourth phase, ensuring they accurately captured the data. This review process resulted in the final four themes used in the findings. In phase five, theme definitions were refined to clarify their scope and conceptual boundaries. The final step involved writing the report and linking themes back to the research questions. Consistent with reflexive thematic analysis [36, 41], we did not aim for data saturation, but instead, prioritised the depth and richness of data.

To enhance trustworthiness, multiple strategies aligned with qualitative best practices were used. These included reflexive note-taking during coding, peer debriefing, and analytic triangulation between team members to check for consistency in theme development. An audit trail was maintained through NVivo 12, and the final theme structure was collaboratively reviewed. These practices supported the credibility, dependability, and confirmability of the findings.

## Results

Four major themes were identified through reflexive thematic analysis: challenges navigating the healthcare system, emotional impact of a CC diagnosis and treatment, the value of specialised cancer nurses and support networks, and physical and mental coping strategies. Each theme encompasses several subthemes, illustrating the nuanced experiences of participants as they navigated their cancer journey. Verbatim quotes are included to ground the findings in participants’ own words and enhance the credibility of the analysis.

### Challenges navigating the healthcare system

A consistent theme across all participants was the challenge of navigating the healthcare system, particularly in the early stages of diagnosis and treatment. Delays, poor communication, and fragmented or uncoordinated care were common experiences, leading to heightened anxiety and stress.

#### Delays in diagnosis and treatment

Several participants described having to actively advocate for themselves to receive timely care. Participant F (Female, 49 years) recounted:

*“I had to fight tooth and nail to even get the procedure done within a timeframe… they were going to make me wait months for a colonoscopy after I’d already lost alitre of blood.”*

Similarly, Participant E (Female, 54 years) stated: *“I had no symptoms, and then one day I’m bleeding, but it still took weeks to get a diagnosis because nobody seemed to be in a rush to figure it out.”*

#### Miscommunication between providers

Another key frustration centred on miscommunication between healthcare providers, which left participants feeling confused and emotionally distressed. Participant F (Female, 49 years) discovered her diagnosis through a hospital cancer nurse before it was officially communicated: *“Nobody had officially told me I had cancer until the [hospital] nurse called and asked how I was coping with it.”* Similarly, Participant A (Female, 41 years) noted:* “You can’t sit back and wait; you’ve got to demand the care and answers you need, otherwise you get lost in the system.”*

#### Inaccessibility and lack of coordinated care

Access to clear communication from hospital-based cancer nurses was also a challenge. Participant H (Male) expressed frustration: *“It’s pretty near impossible to get hold of that bowel cancer nurse when I need to, which is frustrating because there are times when you really need guidance.”* Participant G (Female, 72 years) highlighted how her hospital nurses referred her back to the oncologist without clear answers: *“The oncologist nurses were not all that great with answering my questions. They would always refer me back to the oncologist, but I just needed someone who could give me straightforward answers.”*

Participant A (Female, 41 years) described the distressing experience of receiving conflicting information from different medical teams:

“At first it was, ‘Okay, this is what you have, but you won’t need this.’ Then it changed, ‘We’ll give you chemo and radiation.’ Then I’d get a phone call: ‘No, we’re doing this now.’ And then I’d see the liver doctor, who said, ‘I don’t know why they’re giving you this aggressive chemo.’ That felt like a relief. But then it changed again, ‘We’re going back to you having the port and the chemo.’ It was exhausting- up and down. I just wanted them to have a meeting, discuss my case, and then come to me with a clear plan. Don’t keep meeting and then changing it all.”

These experiences highlight the consequences of poorly coordinated services, limited communication channels, and inadequate continuity of care. Participants emphasised the emotional toll of navigating a fragmented system while already coping with the stress of a cancer diagnosis.

### Emotional impact of a CC diagnosis and treatment

#### Shock and fear following diagnosis

Receiving a diagnosis of CC brought about profound emotional turmoil, with participants frequently describing feelings of shock, fear, and a sense of loss. These emotions were intensified by the suddenness of their diagnosis, particularly for those who had no prior symptoms.

Participant F (Female, 49 years) reflected: *“I had no symptoms before, and suddenly I’m told I have stage three cancer. It was a shock… you just don’t think it can happen to you.”* Similarly, Participant E (Female, 54 years) described the overwhelming shock of being diagnosed with Stage 4 CC cancer without any prior warning: *“I had no symptoms, nothing. For six months, I was fine. Then boom, that was it. It was like a bomb going off.”* Participant B (Female, 41 years) noted how life seemed to change in an instant: *“One day I was fine, and the next I had cancer. There was no warning, nothing. It just hits you like a ton of bricks.”*

#### Fear of recurrence and uncertainty

Participants also expressed fears about treatment efficacy and cancer recurrence. Participant D (Female, 54 years) described this uncertainty: *“It’s the not knowing that kills you—whether the treatment will work, whether it will come back.”*

#### Stigma and isolation

Shame and embarrassment were commonly reported, particularly given the societal stigma that CC cancer can carry compared to more widely recognised cancers such as breast cancer. Participant F (Female, 49 years) stated: *“I felt embarrassed about having bowel cancer… you feel like it’s something you shouldn’t talk about. There’s such a stigma around it, compared to breast cancer.”* Participant G (Female, 72 years) described the emotional disorientation she experienced after chemotherapy ended, leaving her feeling lost: *“Everyone celebrated when I finished chemo, but that was when I felt worst. I didn’t realise how sick I was going to feel afterward, and suddenly there was no routine or next step.”*

#### Support from specialised nurses

Feelings of isolation, both from healthcare providers and families, often exacerbated participants’ distress, with many participants noting that CC cancer receives less visibility and support. For Participant H (Male), emotional support provided by his specialised nurse was crucial:

*“Bowel cancer nurse has been fantastic... she’s been there for me mentally as well as* *physically. She checks in with me regularly, talks me through my concerns, and just* *gives me reassurance. It’s like having a friend who knows exactly what you’re going* *through.”*

These experiences highlight the importance of emotional and psychological support throughout the cancer journey, with specialised nurses providing critical emotional reassurance and practical guidance during these challenging times.

### The value of specialised cancer nurses and support networks

#### Personalised and consistent support

Specialised community cancer nurses helped alleviate some of the emotional burden participants experienced, particularly feelings of isolation and uncertainty. Specialised community cancer care nurses and CC cancer-specific organisations (e.g., Bowel Cancer Australia) played a pivotal role in filling gaps left by the broader healthcare system. Participants frequently highlighted the importance of having consistent, knowledgeable, and empathetic professionals who provided both medical guidance and emotional support. Participant F (Female, 49 years) explained the value of having a consistent point of contact throughout treatment: *“Having a dedicated person who knows your case from start to finish is so important. They understand where you’re coming from, and they can help guide you through it all.”* Similarly, Participant G (Female, 72 years) described how community cancer nurses were more responsive than her hospital nurses, highlighting the value of their expertise and compassionate care:

*“The community cancer nurses were much more able to answer whatever questions I had. I felt more comfortable with them than I did with my oncologist nurse, who couldn’t give me the detailed answers I needed.”*

#### Proactive and compassionate care

Participant A (Female, 41 years) noted the significance of regular check-ins from her CC cancer nurse: *“It made all the difference when the bowel cancer nurse would message me just to check in. Knowing someone was there for me made me feel supported.”* For Participant H (Male), the emotional support provided by the bowel cancer nurse was essential:

*“Bowel cancer nurse has been amazing. She’s seen me on my worst days and checks in even when I don’t reach out. It’s like she knows when I’m having a bad day and just calls at the right time. I don’t know what I would have done without her.”*

#### Holistic and community-based support

Participants praised the holistic approach offered by community cancer support centres, which extended beyond medical care to include services like acupuncture, reflexology, and nutrition advice. This holistic support was critical for many, as Participant E (Female, 54 years) explained: *“They offered me everything—counselling, massages, mindfulness. It wasn’t just about the medical side; they cared for me as a whole person, which made such a difference.”* Participant D (Female, 54 years) echoed this: *“community cancer care nurse wasn’t just about the medical side—it was emotional support, yoga, massages, everything I needed. It was a lifeline for me.”*

#### Support from family and friends

Informal support from family and friends also played a critical role, although it could sometimes present challenges. Participant F (Female, 49 years) described the difficulty of navigating her family’s responses to her diagnosis: *“Sometimes it’s hard with family. They don’t always know what to say, and that can make things harder. But I know they care, and that helps.”* In contrast, Participant C (Female, 54 years) shared how her friends organised fundraisers and stayed connected through regular updates: *“My friends have been amazing… they’ve raised money for my treatment and check in with me constantly.”*

These findings indicate the value of formal and informal support networks in helping individuals cope with the multifaceted challenges of CC cancer. The personalised care, emotional reassurance, and continuity of service provided by specialised nurses were invaluable to participants, who felt that these professionals understood their journey and offered both practical and emotional support.

### Physical and mental coping strategies

#### Mind–body practices

Participants adopted a range of coping strategies to manage the physical and emotional demands of CC treatment. These strategies often combined holistic approaches, such as mindfulness, meditation, physical activity, and dietary changes.

Many participants turned to mind–body techniques to manage stress and maintain level of control. Participant F (Female, 49 years) described: *“I used acupuncture and meditation to help with my anxiety… just to feel like I was doing something to take care of myself.”* Similarly, Participant D (Female, 54 years) emphasised the importance of mindfulness: *“Yoga and mindfulness helped me stay calm and focused. It gave me some sense of control when everything else felt out of my hands.”* Participant G (Female, 72 years) found that acupuncture, combined with engaging in activities like puzzles, provided both physical relief and mental relief:* “Acupuncture was a lifesaver, especially for my nerve pain. And when I wasn’t feeling up for much, I’d do puzzles, they kept my mind occupied and gave me a sense of calm.”*

#### Dietary changes

Dietary changes were also a significant coping mechanism. Participant E (Female, 54 years) became a vegetarian after her diagnosis and felt that adjusting her diet helped her regain a sense of control over her health: *“I completely changed my diet after the diagnosis… I know red meat is linked to bowel cancer, so I wanted to do everything I could to reduce my risk.”*

#### Physical activity

Physical exercise also played an important role in recovery. Participant A (Female, 41 years) shared that staying active helped her regain a sense of normalcy: *“Exercise became really important for me. Even if it was just a walk around the block, it gave me a sense of normality and control.”* For Participant H (Male), maintaining physical activity alongside mindfulness was essential for coping:

*“I’ve been doing meditation regularly, and it’s really helped me manage my emotions. Plus, I’ve kept up with swimming and going to the gym, even when I felt drained. It gave me some sense of control.”*

#### Emotional resilience and reframing

Beyond physical coping mechanisms, participants emphasised the importance of resilience. For many, this involved shifting their perspective on cancer and reframing how they approached their diagnosis. Participant F (Female, 49 years) shared: *“I had to come to peace with this. I didn’t want to think of it as a battle to fight—it just is what it is. That’s the only way I can mentally get through this.”*

These diverse coping strategies helped participants manage the challenges of their cancer journeys while complementing the medical treatments they received.

## Discussion

This study explored the experiences of individuals diagnosed with CC, with a focus on how they navigated the healthcare system, their emotional and informational support needs, and their perceptions of the role of specialised cancer nurses. Through reflexive thematic analysis, four key themes were identified: challenges navigating the healthcare system, emotional impact of a CC diagnosis and treatment, the value of specialised cancer nurses and support networks, and coping strategies used by participants. Below, we discuss each theme in relation to existing literature and highlight recommendations based on participant insights.

Participants voiced frustrations with the healthcare system, particularly in navigating delayed diagnoses, poor communication, and uncoordinated care. Miscommunication between healthcare providers exacerbated feelings of helplessness, especially when critical diagnostic or treatment information was withheld or inconsistently delivered. These experiences align with qualitative research highlighting poor inter-clinician communication and care fragmentation as persistent barriers across CC care settings [42, 43]. Similarly, studies have identified gaps in coordinated care, communication, and timely information-sharing as key challenges following CC diagnosis [43–45]. Prior research has also emphasised the value of specialised cancer nurses in providing medical guidance and emotional reassurance [14]. While this reflects some participants’ experiences in our study, particularly in community-based settings, others described frustration with hospital-based cancer nurses, citing limited access to support and inconsistent communication. These differing accounts highlight variability in nursing support across care settings and suggest that continuity and accessibility remain uneven in practice.

The emotional burden experienced by participants, including shock, fear, and uncertainty, aligns with previous research highlighting the psychological distress common following a cancer diagnosis, particularly when symptoms are absent or the diagnosis is unexpected [46, 47]. In our study, several participants described receiving their CC diagnosis as a sudden and disorienting experience, particularly if they were asymptomatic prior to diagnosis. This emotional shock was often compounded by feelings of stigma. As with previous research, CC stigma has been linked to socially uncomfortable symptoms, such as bowel dysfunction and stoma-related issues [13, 48, 49]. While our participants did not describe specific symptoms in detail, several expressed feeling embarrassed about their diagnosis and noted that CC receives less open discussion than other cancers. These findings are consistent with broader observations that CC is stigmatised more than other cancers, such as breast and prostate cancers, which benefits from greater public awareness and cultural familiarity [45, 50]. Within this context, participants described community-based cancer nurses as playing a valuable emotional support role, particularly when they offered proactive contact and reassurance. This is consistent with research emphasising the importance of consistent emotional support within cancer care settings, particularly when provided by familiar, trusted providers [47].

Support networks, especially from specialised community cancer nurses, emerged as a valued resource for many participants. Participants emphasised the importance of consistent, empathetic professionals who could offer both emotional reassurance and practical assistance across different stages of their care. These findings align with previous research highlighting the central role of specialised nurses in improving communication, emotional wellbeing, and continuity of care in cancer settings [14, 47]. Participants particularly appreciated having a dedicated point of contact, which reduced the burden of retelling their medical history and provided a sense of stability. Some also noted the broader benefits of support from community cancer centres, including allied health and emotional wellbeing services. These findings align with past research that has noted the value of integrated care approaches that extend beyond medical treatment to support the psychological and practical needs of patients [14, 51]. However, these types of services were not uniformly available across participant experiences, highlighting inconsistencies in access to holistic support. This reinforces the need for more consistent implementation of integrated care models in community oncology settings, particularly for those living outside major metropolitan areas [4, 52, 53].

Participants employed various coping strategies to manage the physical and emotional demands of their CC diagnosis and treatment. These included holistic practices such as yoga, mindfulness, and acupuncture, as well as physical exercise and dietary changes. Such strategies were described as ways to regain control and reduce stress during a period of uncertainty. Consistent with previous research, engaging in physical activity was linked to improved mood, a greater sense of routine, and a feeling of regained normalcy [4, 15, 16]. Similarly, dietary changes also emerged as a key coping mechanism. Participants described adapting their diets not only to manage treatment side effects but also to support their emotional wellbeing. This aligns with recent findings highlighting the role of personalised nutritional support in empowering patients and improving cancer care experiences [33]. Our findings emphasise the informal and self-directed ways in which individuals mobilise everyday health behaviours to manage uncertainty and restore a sense of agency. These insights highlight the importance of validating patient-led coping strategies within survivorship care planning.

The study has several limitations. The small sample size may not represent the broader CC population, especially individuals from underrepresented socioeconomic and cultural backgrounds. Most participants in this study identified as female and were in the later stages of cancer, which may have influenced the types of experiences and support needs reported. Additionally, the sample did not include non-binary or gender-diverse individuals, which restricts the study’s ability to speak to the full spectrum of patient identities and perspectives. While qualitative research does not aim for generalisability, acknowledging these omissions is important for improving inclusivity and representation in future research. Self-selection bias may also have been present, with participants potentially more motivated to share their experiences due to particularly positive or negative encounters with the healthcare system. Including caregivers and healthcare professionals in future studies could provide a more comprehensive understanding of the challenges CC patients face across the care continuum. Finally, longitudinal studies would help explore how specialised nursing care and other support services shape survivorship experiences over time.

The findings have several implications for cancer nursing practice and service delivery. Specialised cancer nurses were often described as key supports by participants, particularly when they provided consistent, emotionally attuned care across different phases of treatment. This suggests that ensuring continuity in nursing relationships, not just availability, is vital for building trust and reducing emotional distress. Specialised cancer nurses can also support CC patients by proactively checking in with them, providing clear communication about care processes, and helping connect individuals to other support services (e.g., allied health, peer support). These actions can help reduce fragmentation in care and provide a sense of stability during a disorienting time.

Our findings also highlight the value participants placed on self-directed coping strategies, such as exercise, mindfulness, and dietary changes, and the role nurses can play in validating and supporting these strategies. Specialised cancer nurses working in community settings may be well positioned to provide brief education, referrals, or simply encouragement around patient-led coping approaches. To support equitable access to these benefits, cancer care systems should prioritise consistent models of specialist nursing support across hospital and community settings. In particular, efforts should focus on improving service availability in regional areas, where participants reported greater difficulty accessing emotional and practical support. Strengthening nurse-patient continuity, improving communication pathways, and supporting informal coping efforts may help patients feel less isolated and more empowered across the cancer trajectory.

In conclusion, this study highlights the complex experiences of individuals diagnosed with CC, including difficulties navigating fragmented healthcare systems, the emotional shock of diagnosis, and the value of supportive relationships with healthcare providers. Specialised cancer nurses emerged as key figures when they were consistently available and able to offer both emotional and practical support. However, participants reported variation in access to these services, highlighting a need for greater continuity and availability of nursing support, particularly in community and regional settings. Participants also used self-directed strategies such as exercise, mindfulness, and diet changes to manage the emotional demands of treatment, emphasising the importance of validating and supporting informal coping approaches. Future research should explore how these supports can be made more consistently available and integrated into survivorship care.

## Supplementary Information

Below is the link to the electronic supplementary material.

ESM 1 (DOCX 32.5 KB)

## Data Availability

No datasets were generated or analysed during the current study.
