# Supplementary material for: Experiences of colorectal cancer patients in Australia: a qualitative study on specialised nursing and supportive care
Source: Support Care Cancer. 2026 Jan 29;34(2):140. doi: 10.1007/s00520-026-10333-6 (PMC12852132; doi:10.1007/s00520-026-10333-6)
Supplement: Supplementary file 1 — (DOCX 32.5 KB) [file 520_2026_10333_MOESM1_ESM.docx]

**Supplementary Material – COREQ Checklist and Interview Guide**

**COREQ Checklist (Consolidated Criteria for Reporting Qualitative Research)**

| Domain | Item | Manuscript Evidence |
| --- | --- | --- |
| Research team and reflexivity | Interviewer/facilitator | Yes – explicitly stated: Interviews were conducted by a male member of the research team. |
| Research team and reflexivity | Credentials | Yes – explicitly stated: The interviewer had a background in counselling and graduate-level psychology study. |
| Research team and reflexivity | Occupation | Yes – explicitly stated: The interviewer was working as a counsellor at the time of the study. |
| Research team and reflexivity | Gender | Yes – explicitly stated: Interviewer was male. |
| Research team and reflexivity | Experience and training | Yes – explicitly stated: Interviewer was working as a counsellor at the time of the study, with a background in counselling and graduate-level psychology study. He also received additional training from the co-authors (a clinical psychologist and a psycho-oncology expert) in qualitative interviewing and reflexive thematic analysis prior to data collection.” |
| Research team and reflexivity | Relationship established | Yes – explicitly stated: There was no prior relationship between the interviewer and any of the participants before the study commenced |
| Research team and reflexivity | Participant knowledge of the interviewer | Yes – explicitly stated: Participants were provided with an information sheet outlining the interviewer’s role and the study’s purpose. |
| Research team and reflexivity | Interviewer characteristics | Yes – explicitly stated: Interviewer had a strong interest in patient-centred cancer care and used reflexivity to minimise the influence of prior assumptions |
| Study design | Methodological orientation and theory | Yes – explicitly stated:  Braun and Clarke’s (2006) thematic analysis used; stated explicitly. |
| Study design | Participant selection | Yes – explicitly stated:  Convenience sampling was used; participants self-selected into the study after responding to social media posts or flyers at a community cancer support centre. |
| Study design | Method of approach | Yes – explicitly stated: Participants were recruited via flyers and social media; they contacted the researchers directly via email to participate |
| Study design | Sample size | Yes – explicitly stated: Nine participants took part in the study. |
| Study design | Non-participation | Yes – explicitly stated: There were no refusals or withdrawals; all eligible participants completed the study |
| Study design | Setting of data collection | Yes – explicitly stated: Interviews conducted via phone or Zoom. |
| Study design | Presence of non-participants | Yes – explicitly stated: No individuals other than the participant and interviewer were present during interviews. |
| Study design | Description of sample | Yes – explicitly stated: Table 1 presents age, gender, stage, years since diagnosis, and treatments. |
| Study design | Interview guide | Yes – explicitly stated: Interview guide developed from literature and expert consultation; examples provided in the supplementary materials. |
| Study design | Repeat interviews | Yes – explicitly stated: No repeat interviews were conducted with participants. |
| Study design | Audio/visual recording | Yes – explicitly stated: Interviews were recorded and transcribed verbatim. |
| Study design | Field notes | Yes – explicitly stated: Field notes were not taken; all interviews were audio recorded and transcribed verbatim. |
| Study design | Duration | Yes – explicitly stated: Interviews lasted approximately 40 minutes. |
| Study design | Data saturation | Yes – explicitly stated: Data richness was prioritised over saturation, consistent with reflexive thematic analysis. |
| Study design | Transcripts returned | Yes – explicitly stated: Transcripts were not returned to participants. |
| Analysis and findings | Number of data coders | Yes – explicitly stated: Data were analysed using NVivo 12. |
| Analysis and findings | Description of the coding tree | Yes – partially addressed: Codes and theme development described in narrative using Braun and Clarke’s framework; no formal coding tree presented. |
| Analysis and findings | Derivation of themes | Yes – explicitly stated: Themes derived inductively from codes. |
| Analysis and findings | Software | Yes – explicitly stated: NVivo 12 used for data analysis. |
| Analysis and findings | Participant checking | Yes – explicitly stated: Participants were not asked to review or validate findings. |
| Analysis and findings | Quotations presented | Yes – explicitly stated: Participant quotes used to support themes. |
| Analysis and findings | Data and findings consistent | Yes – explicitly stated: Themes align with presented data and quotes. |
| Analysis and findings | Clarity of major themes | Yes – explicitly stated: Four major themes clearly identified. |
| Analysis and findings | Clarity of minor themes | Yes – some variation within themes was described (e.g., differences in access to support, emotional responses, and coping strategies), but no formal minor themes were labelled. |

# Participant Questionnaire and Interview Guide

## Screening Questions

1. Please indicate your age:

2. Are you currently living in Australia?

3. Are you a current bowel cancer patient or survivor of cancer?

## Demographic Questions

4. What gender do you identify as:
 □ Male □ Female □ Different identity □ Prefer not to say

5. When were you diagnosed with bowel cancer? ___________________________________________

6. Please specify what stage your bowel cancer is at:
 □ Stage 1 □ Stage 2 □ Stage 3 □ Stage 4 □ In remission □ Recovered □ Other: __________

7. If currently undergoing cancer treatment, please specify what type(s):
 □ Surgery □ Chemotherapy □ Radiation □ Immunotherapy □ Hormone therapy □ Targeted therapy □ Other: ___________________

8. If you received cancer treatment in the past, please specify what type(s):
 □ Surgery □ Chemotherapy □ Radiation □ Immunotherapy □ Hormone therapy □ Targeted therapy □ Other: ___________________

## Semi-Structured Interview Guide

Please note that probes will be used in One-on-One Interviews.

1. 1. Can you tell me a bit about your cancer journey/experiences?
2. 2. Tell me about your experiences with Bloomhill and how you got connected with Bloomhill?
3. 3. What have been your experiences with the bowel cancer care nurse specialist? How did you get referred? How has this helped you?
    Probe: What did the nurse specifically do or say that made you feel they had helped.
4. 4. What are the biggest challenges that you have faced (or are facing) from your cancer experience?
    Probe: How have you reacted to these challenges?
    Probe: How have you coped to these challenges?
5. 5. What were (or are) the significant changes in your life due to the diagnosis of bowel cancer? How has it impact work, family, friends, etc.?
6. 6. What about the impact of your cancer on your relationship with your partner? Challenges? Strengths?
7. 7. What coping strategies do you use to deal with issues arising from your cancer?
8. 8. What do you (or someone else facing bowel cancer) need to assist you with the challenges?
9. 9. Are there things that Bloomhill or the Bowel Cancer nurse could do to assist you or others in your situation?
10. 10. Is there anything else you would like to tell me or add about your experiences or challenges with your cancer?
